# Effects of Salinomycin and Deferiprone on Lead-Induced Changes in the Mouse Brain

**DOI:** 10.3390/ijms24032871

**Published:** 2023-02-02

**Authors:** Emilia Petrova, Yordanka Gluhcheva, Ekaterina Pavlova, Ivelin Vladov, Peter Dorkov, Martin Schaier, Irena Pashkunova-Martic, Thomas H. Helbich, Bernhard Keppler, Juliana Ivanova

**Affiliations:** 1 Institute of Experimental Morphology, Pathology and Anthropology with Museum, Bulgarian Academy of Sciences, Acad. Georgi Bonchev Str., Bl. 25, 1113 Sofia, Bulgaria; 2 Chemistry Department, Research and Development, BIOVET JSC, 39 Peter Rakov Street, 4550 Peshtera, Bulgaria; 3 Institute of Analytical Chemistry, University of Vienna, 38 Waehringer Strasse, 1090 Vienna, Austria; 4 Department of Biomedical Imaging and Image-Guided Therapy, Division of Molecular and Structural Preclinical Imaging, Medical University of Vienna and General Hospital of Vienna, 18–20 Waehringer Guertel, 1090 Vienna, Austria; 5 Institute of Inorganic Chemistry, University of Vienna, 42 Waehringer Strasse, 1090 Vienna, Austria; 6 Faculty of Medicine, Sofia University “St. Kliment Ohridski”, Kozjak Str. 1, 1407 Sofia, Bulgaria

**Keywords:** Pb-induced neurotoxicity, mouse brain histology, essential elements, salinomycin, deferiprone

## Abstract

Lead (Pb) is a highly toxic heavy metal that has deleterious effects on the central nervous system. This study aimed to investigate the effects of salinomycin (Sal) and deferiprone (DFP) on brain morphology and on the content of some essential elements in Pb-exposed mice. Adult male Institute of Cancer Research (ICR) mice were exposed to a daily dose of 80 mg/kg body weight ( b.w.) Pb(II) nitrate for 14 days and subsequently treated with Sal (16 mg/kg b.w.) or DFP (19 mg/kg b.w.) for another 14 days. At the end of the experimental protocol, the brains were processed for histological and inductively coupled plasma mass spectrometry (ICP-MS) analyses. Pb exposure resulted in a 50-fold increase in Pb concentration, compared with controls. Magnesium (Mg) and phosphorus (P) were also significantly increased by 22.22% and 17.92%, respectively. The histological analysis of Pb-exposed mice revealed brain pathological changes with features of neuronal necrosis. Brain Pb level remained significantly elevated in Sal- and DFP-administered groups (37-fold and 50-fold, respectively), compared with untreated controls. Treatment with Sal significantly reduced Mg and P concentrations by 22.56% and 18.38%, respectively, compared with the Pb-exposed group. Administration of Sal and DFP ameliorated brain injury in Pb-exposed mice and improved histological features. The results suggest the potential application of Sal and DFP for treatment of Pb-induced neurotoxicity.

## 1. Introduction

Lead (Pb) is a naturally occurring heavy metal that has an adverse impact on human health. Even at low concentrations, Pb is found to affect various organs and systems [1]. Its widespread industrial use has led to increasing human exposure with severe and long-term toxic effects. A variety of factors influence Pb absorption, including the route of exposure, nutritional and health status, and the age of the individual. The absorbed Pb circulates in the blood for approximately 30 days, whereas the half-life of Pb is 2 to 3 years in the brain and about 20–30 years in the bones [1,2,3].

The nervous system is the most vulnerable to Pb-induced toxicity. Pb exposure has been associated with a wide range of neurological disorders, including cognitive deficits, motor and coordination impairment, and behavioral problems [4,5]. The brain barriers—the blood-cerebrospinal fluid (BCSF) barrier and the blood-brain barrier (BBB)—are critical determinants of central nervous system homeostasis and they represent the first target of Pb neurotoxicity [3]. Pb is known to disrupt these barriers’ structural or functional integrity, thus affecting normal brain function [6,7]. The developing brain is particularly sensitive to the deleterious effects of Pb, which is partially attributed to the increased permeability of the brain barriers during this period [4,5,7]. It is reported that infants and children absorb 40–50% of dietary Pb, whereas Pb absorption in adults is only 5–10% [8,9]. Moreover, lead is readily transported across the placenta and breast milk [5].

Lead toxicity is associated with disturbances in the homeostasis of essential elements. Pb exposure is known to affect the levels of calcium (Ca), magnesium (Mg), iron (Fe), and zinc (Zn) in the tissues [10]. Moreover, these effects are found to be different in chronic and acute Pb exposure. Chronic Pb exposure is reported to have a greater impact on the metabolism of macronutrients, essential elements, and neurodegenerative injury [10]. The greatest effect of chronic exposure on essential elements has been reported in the brain, while acute exposure has had the greatest effect on essential elements in the liver [10]. Our previous studies have demonstrated the influence of subacute Pb exposure on the homeostasis of essential elements in mouse kidneys, testes, lungs, and heart [11,12,13]. Disturbances in the levels of essential elements were accompanied by histopathological changes in the target organs.

Different pharmacological strategies have been proven effective against the harmful effect of Pb. Chelation therapy has been used for decades for the treatment of Pb toxicity. We have previously reported data about the effect of the chelating agents meso-2,3-dimercaptosuccinic acid (DMSA), monensin, salinomycin (Sal), and deferiprone (DFP) on cadmium-induced brain injuries in mice [14,15]. Our studies have also demonstrated the effect of these chelators on renal, testicular, lung, and heart toxicity in Pb-exposed mice [11,12,13].

Herein, we present data about the potential of salinomycin and deferiprone to ameliorate Pb-induced damage in the mouse brain with regard to the content of essential elements and histologic pattern.

## 2. Results

### 2.1. Brain Weight Index in Experimental Mice

**Figure 1:**
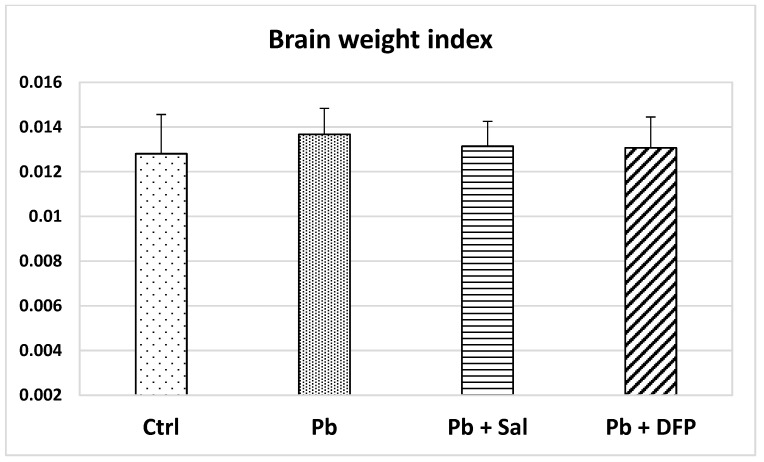
Brain weight index in untreated control mice (Ctrl), Pb-exposed mice (Pb), and Pb-exposed mice treated with salinomycin (Pb + Sal) or deferiprone (Pb + DFP). Data are presented as mean + standard deviation (SD).

Brain weight index (BWI) was calculated as the organ-to-body weight ratio. The lowest value of BWI was found in the untreated control group (Figure 1). No statistically significant differences were found in BWI between the experimental groups.

### 2.2. Pb Concentration in the Brains of Experimental Mice

**Figure 2:**
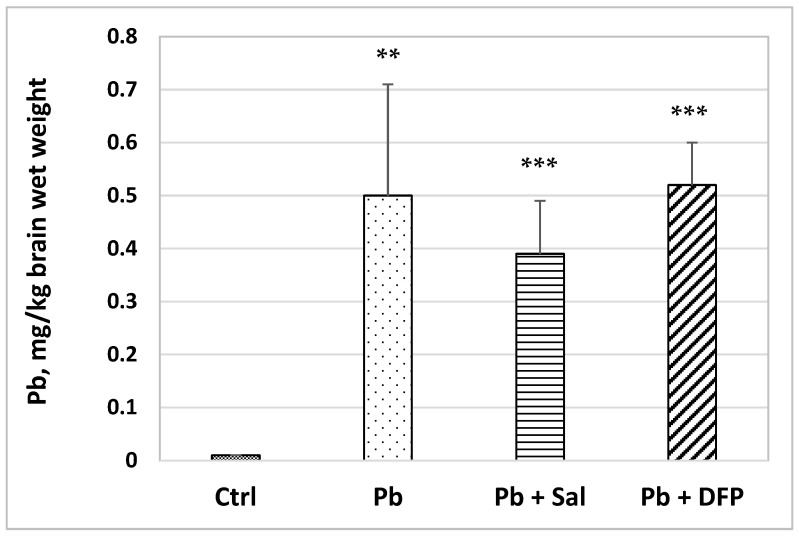
Pb concentration in the brains of experimental mice: Ctrl—untreated control group; Pb—Pb-exposed group; Pb + Sal—Pb-intoxicated mice treated with salinomycin; Pb + DFP—Pb-intoxicated mice treated with deferiprone. Data are presented as mean + SD. ** *p* < 0.01, *** *p* < 0.001 compared with the untreated control group.

The brain Pb concentration was significantly increased in the Pb-exposed mice compared with untreated controls (50-fold, *p* < 0.01) (Figure 2). Pb levels remained elevated in salinomycin-(37-fold, *p* < 0.001) and deferiprone-administered (50-fold, *p* < 0.001) groups compared with the untreated controls. Pb concentration was estimated to decrease by 22% in Sal-treated mice compared with Pb-exposed mice, but the difference was not statistically significant.

### 2.3. Concentrations of Some Essential Elements in the Brains of Experimental Mice

The results for the concentrations of some essential elements in the brains of experimental mice are presented in Table 1. Pb exposure significantly increased magnesium (Mg) and phosphorus (P) concentrations by 22.22% (*p* < 0.05) and 17.92% (*p* < 0.05), respectively, compared with untreated controls. The concentrations of calcium (Ca) and copper (Cu), iron (Fe), and zinc (Zn) were not significantly affected by Pb intoxication.

Treatment with Sal significantly reduced Mg and P concentrations by 22.56% (*p* < 0.01) and 18.38% (*p* < 0.05), respectively, compared with the Pb-exposed group. Compared with untreated controls, Sal administration did not significantly alter the concentrations of Ca, Cu, Fe and Zn.

Treatment with DFP did not significantly affect the concentrations of essential elements in the experimental groups. Mg, P, Ca, Fe, and Cu levels remained higher, compared with the untreated control group.

### 2.4. Brain Histoarchitecture of the Experimental Mice

**Figure 3:**
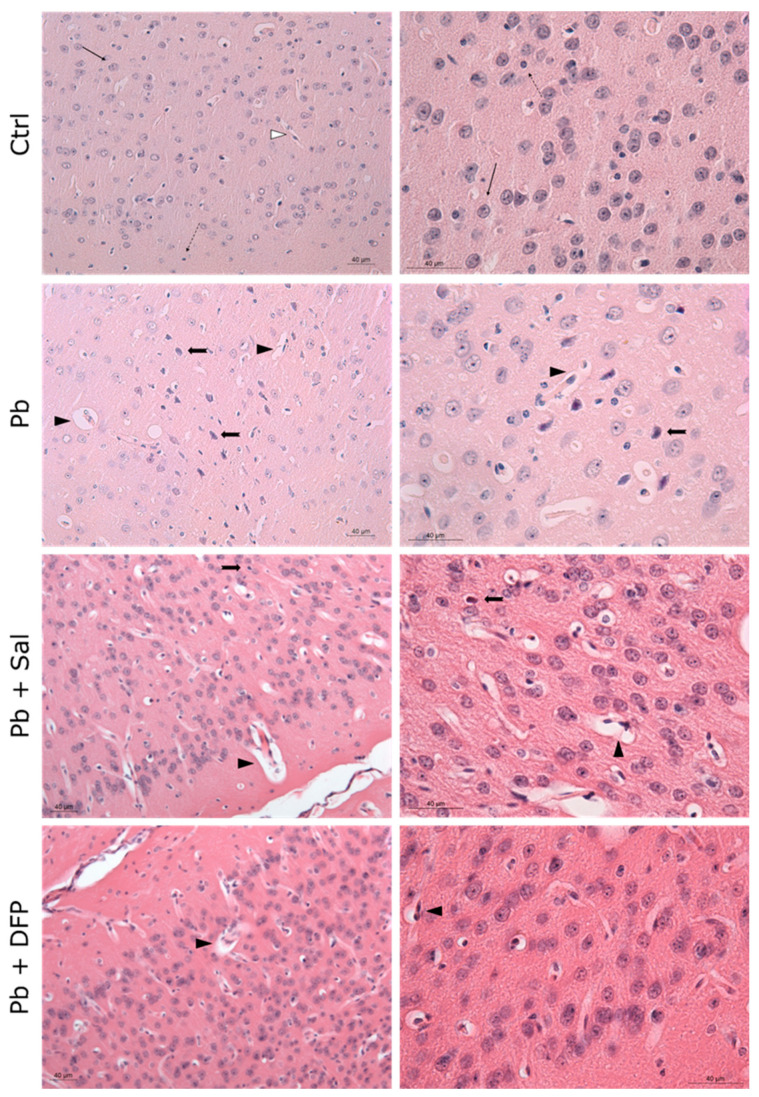
Light microphotographs of the cerebral cortex of experimental mice. Ctrl—untreated control mice. Intact neurons (black, thin arrow), glial cells (black, dotted arrow), and perivascular spaces (white arrowhead). Pb—Pb-exposed group. Numerous pyknotic neurons (black, thick arrow) with prominent perineuronal spaces, dilated perivascular spaces (black arrowhead), and reactive endothelial cells; Pb + Sal—Pb-exposed mice, subjected to treatment with salinomycin. A few pyknotic neurons (black, thick arrow) with prominent perineuronal spaces, dilated perivascular spaces (black arrowhead), and reactive endothelial cells; Pb + DFP—Pb-exposed mice, subjected to treatment with deferiprone. A few dilated perivascular spaces (black arrowhead), and reactive endothelial cells. Hematoxylin-eosin staining, ×200; ×400.

**Figure 4:**
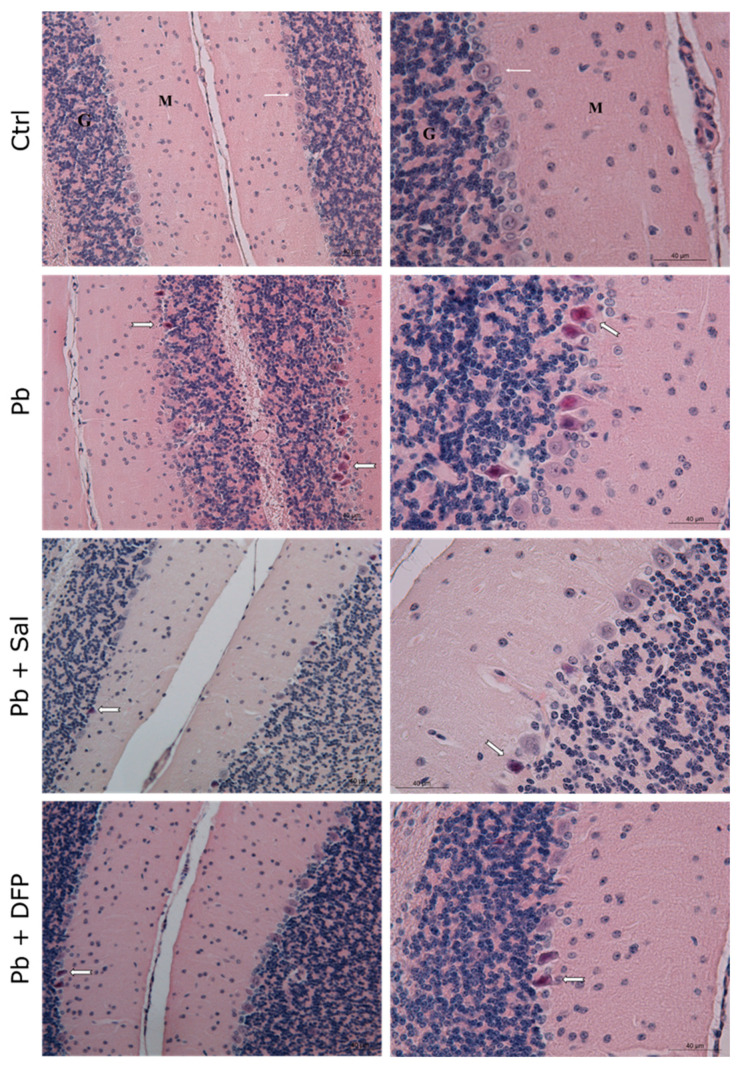
Light microphotographs of the cerebellar cortex of experimental mice. Ctrl—untreated control mice. Different layers of the cortex: molecular layer (M), Purkinje cell layer (white, thin arrow), and granular layer (G). Pb—Pb-exposed mice. Many pyknotic Purkinje cells (white, thick arrow). Pb + Sal—Pb-exposed mice, subjected to treatment with salinomycin. A few pyknotic Purkinje cells (white, thick arrow). Pb + DFP—Pb-exposed mice, treated with deferiprone. A few pyknotic Purkinje cells (white, thick arrow). Hematoxylin-eosin staining, ×200; ×400.

The results of the histological examination of the brain are presented in Figure 3 for the cerebrum and Figure 4 for the cerebellum. Normal histoarchitecture was observed in the cerebral and cerebellar cortices of untreated control mice. The neurons had spherical or pyramidal perikaryon, and round, centrally located nuclei. Morphologically normal glial cells and brain capillaries were also observed. The three layers of the cerebellar cortex were well distinguished: the deep granular layer; the Purkinje cell layer; and the superficial molecular layer. The Purkinje neurons were arranged in a single row and had a large pear-shaped cell body.

Pb intoxication resulted in brain pathological changes with features of neuronal necrosis. Numerous shrunken, darkly stained pyknotic neurons and dilated perivascular spaces were demonstrated in the cerebral cortex. Many damaged, shrunken Purkinje cells with pyknotic nuclei and eosinophilic cytoplasm were seen in the cerebellar cortex. Administration of Sal and DFP ameliorated brain injury in Pb-exposed mice and improved the histological features. Dilated perivascular spaces were still present, but to a lesser extent. Intensive vascularization was observed in both groups treated with chelating agents. Brain histological architecture appeared more similar to that of the controls in DFP-treated mice.

## 3. Discussion

Lead has no known role in biological systems, but it has the capacity to disrupt normal biochemical and physiological functions. Once absorbed in the bloodstream, Pb is distributed throughout the body, accumulating in the soft tissues, such as the brain, liver, and kidney, or in the bones and teeth [5,16]. As shown in our study, Pb exposure at an average daily dose of 80 mg/kg b.w. resulted in a significantly elevated brain Pb concentration (50-fold) compared with untreated mice. Previously, we have reported a significant increase in the testicular, renal, heart, and lung concentrations of Pb in Pb-exposed mice, compared with untreated controls [11,12,13]. Animal studies have indicated that the increases in brain Pb levels depend on the Pb exposure dose [10,17]. The high-dose treatments are found to be accompanied by a slower rate of Pb accumulation due to Pb uptake saturation. Moreover, a dose-dependent effect of Pb on the brain content of essential elements has also been documented [17].

Our results show no significant effect of Pb exposure on the brain weight index when compared with untreated controls. However, other studies have reported a significant reduction in the brain/body weight ratio after Pb treatment [18]. In addition, prenatal Pb exposure may also result in a significant decrease in birth weight, as evidenced by clinical and animal studies [19,20].

Pb is a non-essential element and there are no specific Pb transport proteins/channels. Pb uptake occurs through the essential elements’ transport pathways. The ability of Pb^2+^ ions to interact with oxygen and their high affinity for thiol groups allows Pb^2+^ to substitute or compete with diverse essential divalent cations, such as Ca^2+^, Zn^2+^, Fe^2+^, and Cu^2+^, at the metal-binding sites. This ion-mimicry mode of toxicity is considered responsible for the incorporation of Pb^2+^ into the brain, with the consequent disruption of calcium-dependent signaling pathways, redox and energetic homeostasis, neurotransmission, protein and lipid homeostasis, and neuroinflammation [3].

In our study, brain Mg and P levels were found to be significantly increased (*p* < 0.05) in Pb-exposed mice. Similarly, Yu et al. [17] have reported elevated brain Mg levels in a mouse model of acute Pb exposure. It has been suggested that Pb promotes the removal of Mg^2+^ from the blood to the brain and other organs. Our results in Pb-exposed mice show a slight increase in brain Ca and Cu levels although this was not statistically significant compared with controls. The impact of Pb on calcium metabolism has been suggested as one of the mechanisms underlying Pb neurotoxicity [2,21]. Pb^2+^ easily substitutes Ca^2+^ at the sites of transporters, including Ca^2+^-ATPase, calbindin, protein kinase C, and Ca^2+^ channels. Thus, Pb^2+^ interferes with calcium homeostasis, which leads to an increase in intercellular Ca^2+^. Pb has also been reported to induce Cu accumulation in the brain. Copper is essential for normal brain function as a co-factor for oxidation-reduction reactions, although, excessive Cu^2+^ ions reportedly generate free radicals and cause neurotoxicity [22]. Increased Cu levels have been demonstrated in the choroid plexus of Pb-exposed rats due to morphological changes in the choroid plexus capillary epithelium and altered Cu transporter expression, with a consequent Cu clearance dysfunction [23]. Cu accumulation has also been documented in the striatum and hypothalamus of Pb-exposed rats [24].

Studies have suggested the potential role of divalent metal ion transporter 1 (DMT1) for Pb uptake in the brain [25]. DMT1 is the major iron transporter, which is also involved in the absorption of zinc. The DMT1-mediated Pb transport could explain our data for decreased Fe and Zn levels in the Pb-treated group, as Pb^2+^ competes with Fe^2+^ and Zn^2+^ for DMT1 binding sites. Nutritional deficiencies of trace elements, including Fe, Zn, Ca, and Mg, are shown to increase gastrointestinal Pb absorption, thus enhancing susceptibility to Pb neurotoxicity [3,26].

Our results for the effect of Pb on the levels of essential elements in the brain are supported by prominent histopathological findings in the Pb-exposed mouse brains. Degenerative changes were observed in both the cerebral and cerebellar cortical areas as dilated perivascular spaces and numerous necrotic cortical neurons and Purkinje cells were well demonstrated. These findings are in agreement with other animal studies of Pb neurotoxicity [18,27,28,29]. For example, Sidhu and Nehru [27] have demonstrated disorganization of cells in the cerebral and cerebellar cortical layers in Pb-exposed rats. Khafaf et al. [29] have reported the degeneration and necrosis of rat cortical neurons and Purkinje cells, as well the congestion of blood vessels, neuronophagia, and focal gliosis. Brain edema has been documented in the hippocampus of Pb-exposed rats together with focal gliosis in the cerebrum and cerebellar vacuolization [18]. As has been shown, the prefrontal cortex, cerebellum, and hippocampus are the brain regions most vulnerable to Pb exposure, also evidenced in the developing brain [30]. The degenerative morphological changes in our study could be attributed to oxidative damage. Pb is reported to inhibit antioxidant defense enzymes, such as superoxide dismutase, catalase, and glutathione peroxidase [31,32]. In fact, oxidative stress is considered a key aspect of Pb neurotoxic effects [2,33].

It has been shown that Pb accumulates predominantly in astrocytes where it stimulates inflammatory cytokine secretion with further activation of microglia and macrophages [32,34]. Pb reduces the energy supply from astrocytes to neurons, which might trigger neuronal apoptosis [32]. However, Rahman et al. [35] have found that astrocytes are more susceptible to Pb toxicity than neurons. In contrast, other studies have disclosed the higher sensitivity of neurons to the toxic effects of Pb [34]. Pb has also been reported to accumulate in the choroid plexus and brain endothelial cells, causing an opening of the tight junctions and the loss of epithelial barrier function [6,36].

Various chelators and antioxidants have been approved to remove the toxic Pb^2+^, and their use, either alone or in combination, as has been reviewed in detail by Virgolini and Aschner [3]. However, chelation therapy is the basis for the treatment of metal poisoning. Chelation agents form stable complexes with Pb^2+^, which are easily excreted from the body.

Herein, we compare the effect of two lipophilic chelating agents, salinomycin (administered as tetraethylammonium salt of salinomycin acid) and deferiprone, on Pb-induced brain damage in mice. Salinomycin is a polyether ionophorous antibiotic widely applied in veterinary medicine, and recently recognized as an anticancer agent. Deferiprone is known as a therapeutic agent in chronic iron overload in cases of β-thalassemia.

The administration of salinomycin and deferiprone had no significant effect on the brain weight index compared with the Pb-exposed group and untreated controls. We found that salinomycin reduced brain Pb concentration in Pb-exposed mice, whereas deferiprone caused a slight increase, although these changes did not reach statistical significance. Pb levels remained elevated in salinomycin- (*p* < 0.001) and deferiprone-administered (*p* < 0.001) groups compared with the untreated controls. Similarly, Steinbaugh [37] has estimated a lowered brain Pb concentration in Pb-exposed rats after salinomycin treatment, considering the compound a highly active and selective ionophore for Pb. In contrast to our data, deferiprone has been found effective in mobilizing Pb from the kidney, liver, and spleen in Pb-intoxicated rats [38].

Furthermore, our results revealed that salinomycin significantly reduced brain Mg (*p* < 0.01) and P (*p* < 0.05) concentrations to normal values compared with those in the Pb-exposed group. Unlike our findings, Steinbaugh [37] reported no significant changes in the brain levels of the physiologic elements in Pb-exposed rats after salinomycin administration. We have also found that deferiprone did not significantly affect the concentrations of the essential elements in the brain in the experimental groups, nor the Fe content. Thus, deferiprone was shown to be less effective in restoring the levels of the essential elements in the brain after Pb exposure compared with salinomycin.

The ameliorative effect of salinomycin and deferiprone was well demonstrated in our morphological studies. The chelator agents reduced brain injury in Pb-exposed mice and improved the histological features. Deferiprone was found to have a more pronounced ameliorative effect on Pb-induced brain histopathological changes compared with salinomycin. Available literature data have also demonstrated the protective effect of deferiprone in aluminum-induced brain injury in mice [39].

## 4. Materials and Methods

### 4.1. Chemicals and Reagents

Lead nitrate (Pb(NO_3_)_2_; Pb(II) nitrate), tetraethylammonium hydroxide (Et_4_NOH), and diethyl ether (Et_2_O) were bought from Merck (Darmstadt, Germany). Sodium salt of salinomycinic acid (C_42_H_69_O_11_Na, CAS number: 55721-31-8, purity: >95%) was received from Biovet Ltd. (Peshtera, Bulgaria). Salinomycin acid was synthesized as published in Ivanova et al., 2012 [40]. Results for the purity and spectral characteristics of the compound are given in Ivanova et al., 2012 [40]. Deferiprone (C_7_H_9_NO_2_, CAS number: 30652-11-0, purity: 98%) was purchased from Sigma Aldrich (St. Louis, MO, USA). Nitric acid (HNO_3_) (≥69%, Rotipuran Supra, Carl Roth, Karlsruhe, Germany) and hydrogen peroxide (H_2_O_2_) (30%, Suprapur, Merck, Darmstadt, Germany) were used for digestion of the brains. All samples were diluted with ultrapure water (18.2 MΩ cm, ELGA water purification system, Purelab Ultra MK 2, UK or 18.2 MΩ cm, Milli-Q Advantage, Darmstadt, Germany). An inductively coupled plasma mass spectrometer was calibrated with LabKings standard solutions (Hilversum, The Netherlands).

### 4.2. Animals

Adult male Institute of Cancer Research (ICR) mice were purchased from the experimental and breeding base for laboratory animals—Slivnitza, Bulgaria. The experimental protocol was conducted at the Institute of Experimental Morphology, Pathology and Anthropology with Museum, Bulgarian Academy of Sciences, according to the ARRIVE (Animal Research: Reporting of In Vivo Experiments) guidelines and EU Directive 2010/63/EU for animal experiments [41]. The study was approved by the Bulgarian Agency for Food Safety, Approval number 282 from 24 September 2020.

Mice were kept at standard conditions for light (12 h light/12 h dark cycle), humidity, and temperature (23 ± 2 °C). Each mouse was accommodated in an individual polypropylene cage for precise monitoring of the consumed doses of the compounds. Animals were fed a standard diet and had access to food ad libitum. Mice were allowed to acclimate for one week prior to experiments.

### 4.3. Experimental Design

Forty male, 60-day-old ICR mice, weighing 25–30 g, were randomized into four groups as follows:

Group I—untreated control group (Ctrl, *n* = 10), administered distilled water for 28 days.

Group II—Pb-exposed group (Pb, *n* = 10), subjected to an average daily dose of 80 mg/kg body weight (b.w.) Pb(II) nitrate for 14 days. The compound was administered per os in drinking (distilled) water. From the fifteenth day until day 28 of the experimental protocol, mice received distilled water.

Group III—salinomycin-treated group (Pb + Sal, *n* = 10), initially subjected to Pb(II) nitrate intoxication for 14 days as group II, followed by treatment with tetraethylammonium salt of salinomycinic acid at an average daily dose of 16 mg/kg b.w. for 14 days.

Group IV—deferiprone-treated group (Pb + DFP, *n* = 10), initially subjected to Pb(II) nitrate intoxication for 14 days as group II, followed by treatment with deferiprone at an average daily dose of 20 mg/kg b.w. for 14 days.

The solution intakes were monitored daily and the average daily doses were calculated according to the obtained volumes, when necessary.

The sample size used in this study corresponds to the accepted sample size in toxicological studies.

At the end of the experimental protocol (day 29), animals were sacrificed under light ether anesthesia and brains were excised, weighed, and processed for histological and inductively coupled plasma mass spectrometry (ICP-MS) analyses. The brains of four animals from each group were processed for histological analysis and the brains of six animals from each group were stored at −80 °C for ICP-MS analysis.

### 4.4. ICP-MS Analysis

#### 4.4.1. Sample Preparation

The experimental procedure for digestion and preparation of the samples has been described in our previous manuscripts [11,12]. Briefly, the whole brains were digested in PTF vessels with a mixture of 6 mL 20% HNO_3_ (≥69%, Rotipuran Supra, Carl Roth, Karlsruhe, Germany) and 300 µL H_2_O_2_ (30%, Suprapur, Merck, Darmstadt, Germany). The brain samples were heated on a hot-plate for six hours using a temperature program with a maximum temperature of 200 °C. For full digestion, an additional 2 mL of concentrated HNO_3_ was added and the digestion program was applied one more time. After cooling down, the clear solutions were quantitatively transferred to 15 mL tubes and diluted with Milli Q water to a final volume of 10 mL.

#### 4.4.2. Measurements

The total content of each element in the brains was measured using a single quadrupole ICP-MS Agilent 7800 (Agilent Technologies, Tokyo, Japan) equipped with an Agilent SPS 4 auto sampler (Agilent Technologies, Tokyo, Japan) and a Micro Mist nebulizer at a sample uptake rate of approximately 0.25 mL/min. Quality control was achieved by analysis of the certified reference material, TM-28.4 Lake Ontario water (Environment and Climate Change, Burlington, Canada). More details about the measurement conditions were given in Pavlova et al., 2021 [12] and Gluhcheva et al., 2022 [11].

### 4.5. Histological Analysis

Brains from untreated control and experimental mice were fixed in Bouin fixative for 24 h and paraffin-embedded. Briefly, after fixation, the samples were dehydrated in a graded series of ethanol, cleared with xylene, impregnated in molten paraffin, embedded in fresh molten paraffin, and cut into 5-μm-thick coronal sections using a microtome Leica RM2125RTF (Leica Microsystems, Buffalo Grove, IL, USA). Subsequently, sections were stained with hematoxylin and eosin (HE) and observed on a light microscope Leica DM 5000B (Leica Microsystems, Wetzlar, Germany).

### 4.6. Statistical Analysis

All experimental results are presented as mean ± SD. The statistically significant difference between the groups was calculated by one-way analysis of variance (ANOVA) with a Tukey’s post hoc test. A Student’s *t*-test was also applied to estimate statistically significant differences between the two means. The statistical analysis of the experimental data was performed by SPSS package PASW Statistics 23.

## 5. Conclusions

In this study, we present, for the first time, comparative data for the effect of salinomycin and deferiprone on Pb-induced damage in the mouse brain. We have demonstrated a better ameliorative effect of salinomycin on the content of essential elements, whereas deferiprone was found to be more effective in restoring the histological pattern in the brain. The results improve our knowledge of the ability of both chelating agents to counteract Pb-induced neurotoxicity. Nevertheless, further studies are needed to fully characterize the potential of salinomycin and deferiprone for therapeutic use.

## Figures and Tables

**Table 1:** Concentrations of essential elements in the brains of experimental mice. Data are presented as mean + SD.

Group	Mg, mg/kg	P, mg/kg	Ca, mg/kg	Fe, mg/kg	Cu, mg/kg	Zn, mg/kg
**Ctrl**	115.50 ± 12.02	1506.63 ± 139.09	51.44 ± 12.35	24.23 ± 2.61	4.07 ± 0.46	18.04 ± 5.07
**Pb**	141.16 ± 12.09 *	1776.62 ± 126.92 *	55.01 ± 3.61	22.28 ± 2.00	4.41 ± 0.44	15.85 ± 0.91
**Pb + Sal**	109.31 ± 12.11 ^a^	1450.03 ± 164.17 ^a^	53.63 ± 8.39	27.11 ± 3.65	4.04 ± 0.70	14.75 ± 2.13
**Pb + DFP**	138.65 ± 35.34	1789.72 ± 395.87	65.61 ± 16.99	29.03 ± 6.91	4.79 ± 1.18	17.70 ± 4.04

* *p* < 0.05—significant difference compared with the untreated control group; ^a^—significant difference compared with the Pb-exposed group, *p* < 0.05.

## Data Availability

All data necessary to understand or reproduce this study are included in the manuscript.
